# Exploring Treatments for a Rare Guillain-Barré Variant: A Case Report of Miller-Fisher Syndrome

**DOI:** 10.7759/cureus.65561

**Published:** 2024-07-28

**Authors:** Pradnya M Diggikar, Tushar Pancholi, Bhavya Yammanuru, Mayank Mundada, Janani R.

**Affiliations:** 1 Internal Medicine, Dr. D. Y. Patil Medical College, Hospital and Research Centre, Dr. D. Y. Patil Vidyapeeth, Pune (Deemed to be University), Pune, IND

**Keywords:** plasmapheresis, ophthalmoplegia syndrome, intravenous immunoglobulins (ivig), gbs variant, miller-fisher syndrome

## Abstract

The symptoms of Miller-Fisher syndrome (MFS) are a triad of areflexia, ataxia, and ophthalmoplegia. The condition is a rare variant of Guillain-Barré syndrome (GBS), an acute immune-mediated nerve disorder. Both conditions involve abnormal autoimmune responses that may often be triggered by infections such as *Campylobacter jejuni*, human immunodeficiency virus, Epstein-Barr virus, and Zika virus, among others. As a result, the immune system mistakenly attacks the body's own nerve tissues.

MFS is characterised by ophthalmoparesis, which can progress to complete external ophthalmoplegia and may include ptosis, facial nerve paralysis, sensory impairments, and muscle weakness. Diagnosis is supported by lumbar puncture, revealing albumin-cytologic dissociation, although initial tests may not always be indicative. A diagnostic marker for MFS is the presence of anti-GQ1b antibodies, which target the GQ1b ganglioside in nerves and affect oculomotor function in particular. Electrodiagnostic studies often show absent or reduced sensory responses without reduced conduction velocity. Treatment options include intravenous immunoglobulin therapy and plasmapheresis, which are both equally effective.

This case study demonstrated significant clinical improvement in a patient undergoing plasmapheresis due to financial constraints, highlighting the efficacy of this treatment approach. A 50-year-old female presented with limb paraesthesia, progressive ptosis, imbalance, and transient diplopia following a recent fever. Examination revealed stable vitals, decreased deep tendon reflexes, reduced vibratory sensation, cerebellar ataxia, and cranial nerve abnormalities. Cerebrospinal fluid analysis showed elevated protein, suggesting MFS. Normal magnetic resonance imaging and nerve conduction studies indicated GBS, with positive anti-GQ1b antibodies. After five plasma exchange cycles, the patient improved substantially and was discharged with no residual symptoms after one month.

## Introduction

Miller-Fisher syndrome (MFS) is a rare neurological disorder that was first characterised by Miller-Fisher in 1956 as a combination of ataxia, areflexia, and ophthalmoplegia [1]. It is an uncommon type of Guillain-Barré syndrome (GBS), a group of sudden-onset nerve disorders triggered by the immune system [2]. Whereas GBS affects approximately one or two individuals per 100,000 annually, MFS is rarer, with a frequency of about one to two cases per million annually [3]. This syndrome is typically precipitated by bacterial or viral infections that cause the immune system to mistakenly attack nerve tissues. The resulting symptoms include ophthalmoparesis, facial nerve paralysis, and sensory impairments.

Diagnostic procedures often involve lumbar puncture and the detection of anti-GQ1b antibodies; these antibodies are present in most MFS cases. Treatment primarily consists of intravenous immunoglobulin therapy or plasmapheresis, both of which are effective. This discussion focuses on the clinical features, diagnostic challenges, and treatment options for MFS. The paper presents a case study of a specific patient.

## Case presentation

**Figure 1:**
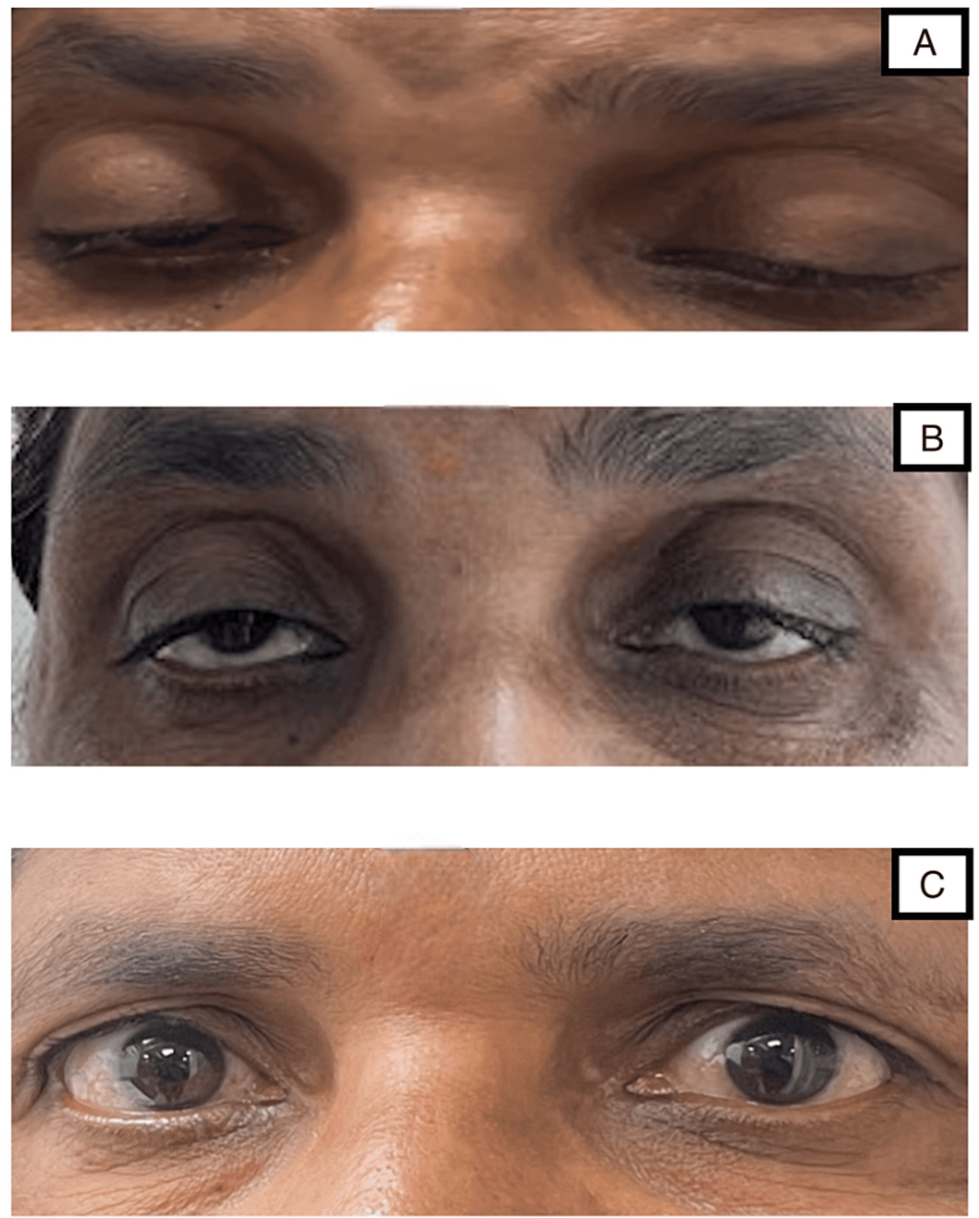
A) Ptosis of both eyes on presentation; B) Improvement of ptosis after 15 days; C) Improvement of ptosis after 30 days

A 50-year-old woman presented to the Outpatient Department with complaints of bilateral upper limb paraesthesia, extending from hand to elbow, and lower limb paraesthesia extending from the foot to knee. She had also experienced ptosis of the right eye for the past day, which progressed to the left eye on the day after admission (Figure 1A). She reported an imbalance in walking since the previous day and transient diplopia since the previous day. She gave no history of any similar incidents in the past. There was no reported history of difficulty in chewing, dysphagia, neck holding, or muscle weakness, and no diurnal variation in ptosis and fatigability. She had a history of fever two weeks ago, which had subsided by now. She had no other significant comorbidity. 

On examination, her pulse rate was 110 bpm, blood pressure was 110/80 mmHg, respiratory rate was 14 per minute, and oxygen saturation was 98% on room air. Clinical neurological examination revealed that the power and tone of the bilateral upper limbs and lower limbs were normal. Deep tendon reflexes were absent in the bilateral biceps, triceps, knee, and ankle, and she displayed decreased sensation of vibrations in the bilateral lower limbs up to the tibial tuberosity. The patient had cerebellar ataxia, leading to a wide-based gait on examination. 

**Figure 2:**
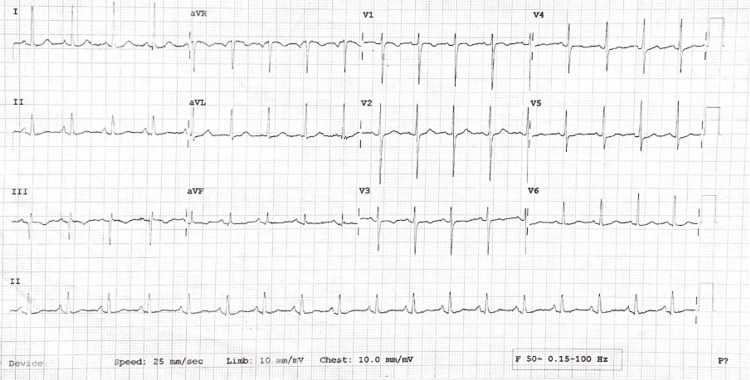
Electrocardiogram showing sinus tachycardia

**Figure 3:**
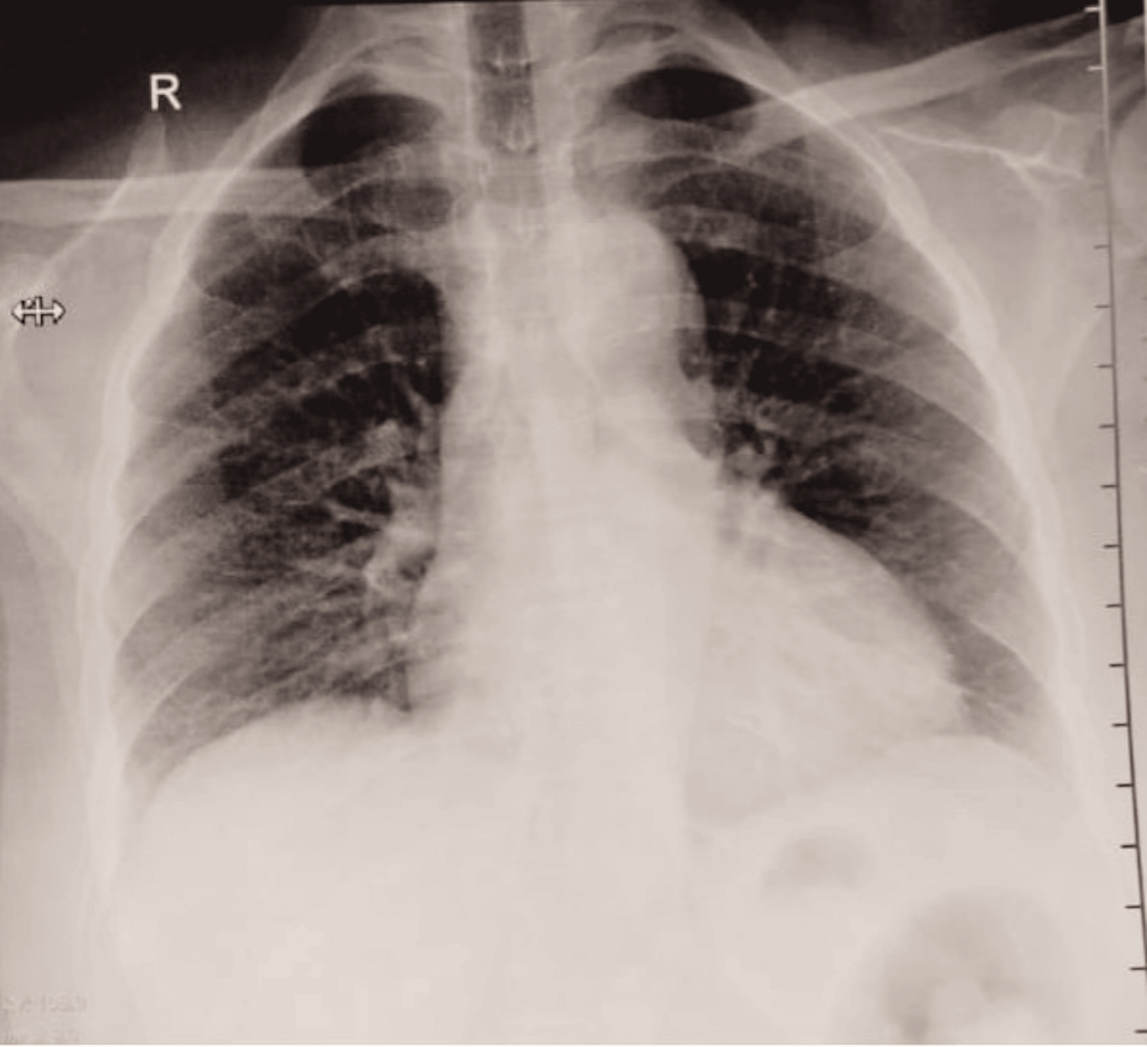
Chest X-ray posteroanterior view, showing no obvious abnormality

Her pupils were bilaterally reactive to light. Cranial nerve examination revealed an abnormal left sixth and bilateral third nerve. All others were within normal limits. Respiratory, cardiovascular, and abdominal examinations were unremarkable, although the electrocardiogram (ECG) showed sinus tachycardia (Figure 2). The chest X-ray of the patient revealed no obvious abnormality (Figure 3).

Laboratory investigations revealed no obvious abnormality on presentation (Table 1).

**Table 1:** Laboratory investigations T3: Triiodothyronine; T4: Thyroxine; TSH: Thyroid stimulating hormone

Investigations	Values	Normal values
Haemoglobin	13 gm/dL	13.2-16.6 gm/dL
Total leukocyte count	15,100 mcL	4,000-10,000 mcL
Platelet	178,000/mm^3^	150,000-410,000/mm^3^
Total bilirubin	0.75 mg/dL	0.22-1.2 mg/dL
Direct bilirubin	0.26 mg/dL	Up to 0.5 mg/dL
Aspartate transaminase	14 U/L	8-48 U/L
Alanine transaminase	13 U/L	7-55 U/L
Urea	21 md/dL	17-49 mg/dL
Creatinine	0.68 mg/dL	0.6-1.35 mg/dL
Serum sodium	138 mEq/L	136-145 mEq/L
Serum potassium	4.11 mEq/L	3.5-5.1 mEq/L
T3	0.96 ng/mL	0.64-1.52 ng/mL
T4	9.56 mcg/dL	4.87-11.72 mcg/dL
TSH	0.87 mcIU/mL	0.35-4.97 mcIU/mL

Based on the findings, a differential diagnosis of MFS was considered, prompting a cerebrospinal fluid (CSF) examination. The CSF analysis revealed a total leukocyte count of 10 leukocytes/mm³, a glucose level of 0.7 g/L, and a protein level of 0.75 g/L (Table 2). The corresponding blood glucose level was 100 mg/dL. The CSF results supported our findings, and a clinical diagnosis of MFS was confirmed.

**Table 2:** Analysis of cerebrospinal fluid RBC: Red blood cells; TLC: Total leukocyte count

CSF examination	Patient's values	Normal values
Physical		
Quantity	2 mL	-
Appearance	Clear, transparent	-
Cobwebs or coagulum	Absent	Absent
Deposits	Absent	Absent
Chemical		
Proteins	68 mg/dL	15-45 mg/dL
Glucose	50 mg/dL	40-80 mg/dL
Microscopy		
RBC	Absent	Absent
TLC	10/cu.mm	0-5/cu.mm
Polymorphs/neutrophils	0%	-
Lymphocytes	100%	-

A magnetic resonance imaging brain scan was conducted, and the results indicated no obvious abnormality. Nerve conduction velocity studies were performed, revealing intermittent F waves in the left median nerve and absent F waves in the bilateral tibial nerve and right peroneal nerve, with sural nerve sparring. These findings strongly indicate a diagnosis of GBS. A GBS antibody profile was thus performed and showed anti-GQ1b antibody positive (Table 3).

**Table 3:** Anti-GQ1b antibody test

Investigation	Observed value	Normal value
Anti-GQ1b antibodies	126 (strong positive)	0-7: negative; 8-14: borderline; 15-70: positive; 71-255: strong positive

The patient was immediately scheduled for plasma exchange, and a total of five cycles were completed. Her neurological symptoms improved progressively, and she was discharged from the hospital after eight days. She was advised to attend regular follow-ups. She showed no residual neurological weakness after one month, and her ptosis had resolved completely during this same period (Figures 1B-1C).

## Discussion

A syndrome that was initially referred to as "the syndrome of ataxia, areflexia, and ophthalmoplegia" was renamed following its description by Miller-Fisher in 1956 [1]. MFS is today identified as an uncommon form of GBS, with GBS being a collection of sudden-onset nerve disorders that are triggered by the immune system [2]. GBS occurs globally at a rate of roughly one or two cases per 100,000 people, whereas the MFS variant is a significantly smaller subset, with an incidence of about one to two cases per million individuals [3]. The MFS variant is characterised by autoimmune activity in which the body's immune system mistakenly targets the nerves. While specific treatments exist, most cases require management to alleviate the symptoms [4].

The onset of MFS can be precipitated by specific viral or bacterial infections present in the environment or food. In response to these infections, the body mistakenly identifies nerve tissues as foreign due to similarities in the protein structures. The result is nerve damage and a subsequent manifestation of symptoms. *Campylobacter jejuni* is frequently implicated as the primary bacterial trigger for both GBS and MFS, often associated with symptoms such as abdominal pain and diarrhoea. Infection by viral pathogens, including human immunodeficiency virus, Epstein-Barr virus, and Zika virus, has also been linked to instances of MFS and GBS [4].

Ophthalmoparesis in MFS, often affecting both eyes, progresses to complete external ophthalmoplegia within one to two weeks. Areflexia is a less consistent component of the triad and is absent in about 18% of cases, or it may also be localised to a specific region of the body. Additionally, this symptom trio is frequently accompanied by supplementary signs such as ptosis (observed in 60% of cases), facial nerve paralysis (30-50%), sensory impairments (20-50%), and muscle weakness (20-25%) [2].

When GBS or MFS is suspected, a lumbar puncture is typically performed to help narrow down the potential diagnosis. A key indicator of GBS and MFS is albumin-cytologic dissociation, which is characterised by a normal cell count alongside raised protein levels in the CSF; this pattern is evident in nearly 90% of patients at the disease’s peak. However, it is important to note that only 50% of patients display albumin-cytologic dissociation upon initial analysis. In addition, a normal protein level, particularly in the early stages of the disease, does not necessarily exclude the diagnosis [3].

The patient in our case study tested positive for anti-GQ1b antibodies. These antibodies target the GQ1b ganglioside component found in nerves and are detected in approximately 85-90% of individuals with MFS. Moreover, the presence of the GQ1b antibody is closely linked to lesions that affect the oculomotor nerves, which explains its association with pronounced oculomotor weakness observed in patients with GBS as well as MFS [5]. Several researchers have proposed that antibodies that target gangliosides - specifically the IgG anti-GQ1b antibody - are a distinctive characteristic of MFS [3,6,7]. The development of ophthalmoparesis in MFS evidently stems from the direct impact of Anti-GQ1b antibodies on the neuromuscular junction that connects the cranial nerves to the muscles controlling eye movement [3]. Electrodiagnostic studies or nerve conduction studies may show reduced or absent sensory responses [3]. By contrast, sensory conduction studies performed at the same time show no evidence of slowing [3].

Treatment of MFS includes mainly intravenous immunoglobulin therapy (IVIg) or total plasma exchange (TPE) [8]. Pinto et al. [9] conducted a systematic review of 20 studies to compare overall IVIg and TPE treatments for GBS, assessing the treatment efficacy with established disability scales such as the Hughes and GBS disability scales. The review also considered functional independence measures and Montebello rehabilitation factor scores. Six randomised clinical trials were reviewed that had been conducted mainly in the 1990s, with participant numbers ranging from 24 to 379. Among them, four trials found IVIg and TPE equally effective. IVIg was deemed as effective as TPE, or more so, for patients aged four years and older [9]. 

Notably, however, the TPE group experienced longer median treatment delays than the IVIg group, and lower complication rates were observed with IVIg treatment [9]. Although the rates of clinically adverse events were similar, IVIg had fewer treatment-related adverse effects than TPE. Only one trial differentiated between disease-specific and treatment-specific adverse events among GBS patients. In patients who tested positive for antiganglioside antibodies, IVIg treatment led to faster clinical recovery compared to TPE, suggesting that double plasma filtration may reduce the efficacy of TPE [9]. Nine additional studies, including four prospective and five retrospective ones, concluded that both TPE and IVIg were viable GBS treatment options [9]. 

Some studies indicated that TPE was marginally more effective than IVIg. While the overall safety profiles were acceptable, one study reported higher complication rates with TPE than with IVIg. Two studies involving Egyptian children favoured TPE treatment, with 95% of IVIg-refractory patients who tested positive for antiganglioside antibodies showing a positive response to TPE. Additionally, shorter hospital stays and higher recovery rates were reported with TPE compared to IVIg treatment [9]. A study by Zaki et al. compared the two therapies and showed insignificant differences in the curative effect of IVIg and TPE [10]. 

In our case study, financial constraints led to the patient being treated with TPE. She showed significant improvement after every follow-up visit.

## Conclusions

Early diagnosis of MFS through clinical examination is crucial for effective disease management and improved patient outcomes. Recognising the characteristic triad of ophthalmoparesis, ataxia, and areflexia, along with supplementary symptoms such as ptosis and facial nerve paralysis, allows clinicians to promptly identify MFS. Timely diagnosis enables the initiation of treatments such as IVIg or TPE. Prompt treatment prevents the progression of symptoms and reduces the risk of severe complications, such as respiratory paralysis or bulbar palsy.

In our case study, the patient's significant improvement following TPE treatment underscores the importance of early and accurate clinical diagnosis in effectively managing MFS. Even with complete treatment, recovery can take approximately 30 days due to the time required for the remyelination of nerve fibres. Such remyelination allows for resolution without any residual neurological deficit, as was observed in our patient.

## References

[1] Fisher M (1956). An unusual variant of acute idiopathic polyneuritis (syndrome of ophthalmoplegia, ataxia and areflexia). N Engl J Med.

[2] Noioso CM, Bevilacqua L, Acerra GM (2023). Miller Fisher syndrome: an updated narrative review. Front Neurol.

[3] Kondrat’ev SA, Kondrat’eva EA, Ternovykh IK, Alekseeva TM, Nazarov RV, Kondrat’ev AN, Ulitin AY (2023). Miller Fisher syndrome. Russ J Anesthesiol Reanimatol.

[4] (2024). Miller Fisher syndrome - symptoms, causes, treatment. https://rarediseases.org/rare-diseases/miller-fisher-syndrome/.

[5] Kumar Gupta S, Kishor Jha K, Diaa Chalati M, Tareq Alashi L (2016). Miller Fisher syndrome. BMJ Case Rep.

[6] Kuwabara S (2016). Pathophysiology of ataxia in Fisher syndrome (Article in Japanese). Brain Nerve.

[7] Marziali S, Picchi E, Di Giuliano F (2017). Acute disseminated encephalomyelitis following Campylobacter jejuni gastroenteritis: case report and review of the literature. Neuroradiol J.

[8] (2024). Miller Fisher syndrome. https://my.clevelandclinic.org/health/diseases/24138-miller-fisher-syndrome.

[9] Pinto AA, De Seze J, Jacob A, Reddel S, Yudina A, Tan K (2023). Comparison of IVIg and TPE efficacy in the treatment of neurological disorders: a systematic literature review. Ther Adv Neurol Disord.

[10] Zaki HA, Iftikhar H, Najam M (2023). Plasma exchange (PE) versus intravenous immunoglobulin (IVIG) for the treatment of Guillain-Barré syndrome (GBS) in patients with severe symptoms: a systematic review and meta-analysis. eNeurologicalSci.

